# Machine learning‐based early prediction of asthma in preschoolers: The COCOA birth cohort study

**DOI:** 10.1111/pai.70223

**Published:** 2025-10-17

**Authors:** Chang Hoon Han, Seok‐Jae Heo, Haerin Jang, So‐Yeon Lee, Ji Soo Park, Dong In Suh, Youn Ho Shin, Jihyun Kim, Kangmo Ahn, Myung Hyun Sohn, Eom Ji Choi, Sun Hee Choi, Hey‐Sung Baek, Soo‐Jong Hong, Kyung Won Kim, Inkyung Jung, Soo Yeon Kim

**Affiliations:** ^1^ Department of Biomedical Systems Informatics Yonsei University College of Medicine Seoul Korea; ^2^ Yonsei Institute for Digital Health Yonsei University Seoul Korea; ^3^ Division of Biostatistics, Department of Biomedical Systems Informatics Yonsei University College of Medicine Seoul Korea; ^4^ Department of Pediatrics Severance Hospital, Yonsei University College of Medicine Seoul Korea; ^5^ PHI Digital Healthcare, Institute for Innovation in Digital Healthcare Yonsei University Seoul Korea; ^6^ Department of Pediatrics Seoul National University College of Medicine Seoul Korea; ^7^ Department of Pediatrics Kyung Hee University College of Medicine Seoul Korea; ^8^ Department of Pediatrics Samsung Medical Center, Sungkyunkwan University School of Medicine Seoul Korea; ^9^ Department of Pediatrics CHA Gangnam Medical Center Seoul Korea; ^10^ Department of Pediatrics Kyung Hee University Hospital at Gangdong, Kyung Hee University College of Medicine Seoul Korea; ^11^ Department of Pediatrics Kangdong Sacred Heart Hospital, Hallym University School of Medicine Seoul Korea; ^12^ Department of Pediatrics, Humidifier Disinfectant Health Center National Medical Center Seoul Korea

**Keywords:** asthma, birth cohort, child, machine learning, preschool

## Abstract

**Background:**

Early prediction of asthma in preschoolers, which is crucial for timely intervention, remains challenging. This study aimed to develop a machine learning (ML)‐based model and a questionnaire‐based scoring tool for the prediction of asthma at age 3 years.

**Methods:**

Data from the COhort for Childhood Origin of Asthma and allergic diseases (COCOA), a comprehensive prospective birth cohort in South Korea, was used. Children with complete 3‐year follow‐up (*n* = 2007) were divided into development (*n* = 1472) and validation (*n* = 535) cohorts based on birth year. Asthma diagnosis at age 3 years was based on physician diagnosis, recurrent wheezing episodes, asthma treatment, or parental reports. Random Forest‐based predictive models were developed using data collected until the age of 2 years, initially selecting features via least absolute shrinkage and selection operator (LASSO) regression. A questionnaire‐based scoring tool was also developed and compared with multiple ML algorithms.

**Results:**

The ML‐based prediction models showed improved performance as the data accumulated. The 6‐month, 1‐year, and 2‐year models had area under the receiver operating characteristic curve (AUROC) values of 0.614, 0.726, and 0.774, respectively, in the validation cohort. The performance of the questionnaire‐based scoring tool (AUROC, 0.790) was comparable to that of the ML‐based model. Important predictors included paternal total IgE levels, maternal iron supplementation during pregnancy, parental asthma history, nut allergy history, and recent lower respiratory infections.

**Conclusions:**

Our study successfully developed robust predictive models for early asthma that demonstrated high performance. The questionnaire‐based scoring tool offers particular value because of its clinical applicability. Further validation in diverse populations and investigation of the causative pathways of the identified predictors are necessary to enhance clinical utility.

**Key message**

This study presents validated machine learning models and a questionnaire‐based scoring tool for predicting asthma at age three using comprehensive birth cohort data. Both approaches demonstrated strong performance, identifying practical predictors such as parental asthma, nut allergy, and early respiratory infections, while maternal iron supplementation was associated with reduced risk. The questionnaire tool's comparable accuracy to machine learning models incorporating advanced examinations highlights its practicality for routine clinical use, offering a scalable and generalizable strategy for early risk stratification and preventive care in pediatric asthma.

## 1 INTRODUCTION

The global burden of asthma in children and adolescents is substantial. As the most common non‐communicable disease in childhood, asthma affects approximately 10% of the pediatric population worldwide.^1^, ^2^ However, its clinical trajectory is highly heterogeneous, ranging from early remission to lifelong persistence.^3^ Although more than half of the children who eventually develop persistent asthma report symptom onset before the age of three, distinguishing them from transient early wheezers remains difficult.^4^, ^5^ Consequently, the early prediction of childhood asthma remains a major challenge.

Over the years, numerous attempts have been made to predict childhood asthma, starting with clinical scoring systems and evolving toward machine learning (ML)‐based techniques.^6^, ^7^ Beginning with the Asthma Predictive Index and its modifications, early efforts focused on scoring systems and regression‐based models targeting different age groups and prediction windows.^6^, ^7^, ^8^, ^9^, ^10^ However, these conventional models have demonstrated suboptimal predictive accuracy, inconsistent performance, and limited applicability in real‐world clinical settings.^6^, ^9^, ^10^ More recently, ML approaches have shown promise in overcoming some of these limitations by capturing complex, nonlinear interactions among a wide range of risk factors.^11^, ^12^, ^13^ Data‐driven approaches leveraging longitudinal birth cohort data—incorporating early‐life exposures, symptom trajectories, and environmental factors—have enabled the development of more accurate predictive models. Moreover, these models have facilitated the identification of novel early‐life determinants of asthma.^11^, ^13^

In this study, we aimed to develop a predictive model for early childhood asthma using data from the COhort for Childhood Origin of Asthma and allergic diseases (COCOA), an ongoing prospective population‐based birth cohort study established in South Korea in 2007.^14^, ^15^ COCOA follows mother–child pairs from the prenatal period onwards, collecting high‐resolution longitudinal data, including data on environmental exposures, dietary patterns, symptom histories, clinical assessments, and laboratory findings. This comprehensive dataset provides an optimal foundation for building robust ML‐based models for early risk prediction.

Furthermore, we aimed to develop a simplified scoring tool using only questionnaire‐derived information from the COCOA database. Although incorporating laboratory and biomarker data is known to enhance model performance, such requirements may limit feasibility in real‐world clinical settings.^6^ By restricting predictors to easily obtainable questionnaire items, we aimed to create a low‐cost, accessible tool that maintains strong predictive performance.

To the best of our knowledge, this is the first study to develop and validate a questionnaire‐only tool, based on rich birth cohort data, that retains a meaningful predictive value while ensuring broad applicability in routine clinical settings.

## 2 MATERIALS AND METHODS

### 2.1 Study Participants

The rationale and methodology of the COCOA birth cohort study have been previously described.^14^, ^15^ For the current study, we included participants who had completed follow‐up assessments up to the age of 3 years by the end of 2020. To enhance generalizability, participants born before 2015 were assigned to the development cohort and those born in or after 2015 to the validation cohort, simulating a temporal validation framework with later‐born children used for validation.^16^

The COCOA study was approved by the Institutional Review Boards of each participating center: Asan Medical Center (IRB No. 2008–0616), Samsung Medical Center (IRB No. 2009‐02‐021), Severance Hospital (IRB No. 4‐2008‐0588), CHA Medical Center (IRB No. 2010–010), and Seoul National University Hospital (IRB No. H‐1401‐086‐550).

### 2.2 Definitions

The presence of asthma at the age of 3 years was ascertained based on either information retrieved from physician records or parent‐reported questionnaires (Table S1). Physician‐based criteria included a diagnosis of asthma, history of recurrent wheezing, diagnosis of bronchiolitis with more than two episodes, or documented asthma treatment. Questionnaire‐based criteria included parent‐reported wheezing with more than two episodes in the past year, a prior physician diagnosis, or asthma treatment. Children who met any of these criteria were classified as having asthma. Controls were defined as study participants with no history of diagnosis or treatment for asthma, atopic dermatitis, allergic rhinitis, or food allergies. This definition was chosen to maintain a clear distinction between cases and controls, as children who had developed other allergic diseases may represent an intermediate‐risk group for asthma rather than a truly unaffected control population.

### 2.3 Data Preprocessing & Feature Selection

For participants included in the model development, data collected up to the age of 2 years were utilized. Variables with more than 80% missing data were excluded from the analysis. Missing values for the remaining variables were imputed using the MissForest algorithm, a non‐parametric method based on Random Forest.^17^

**FIGURE 1:**
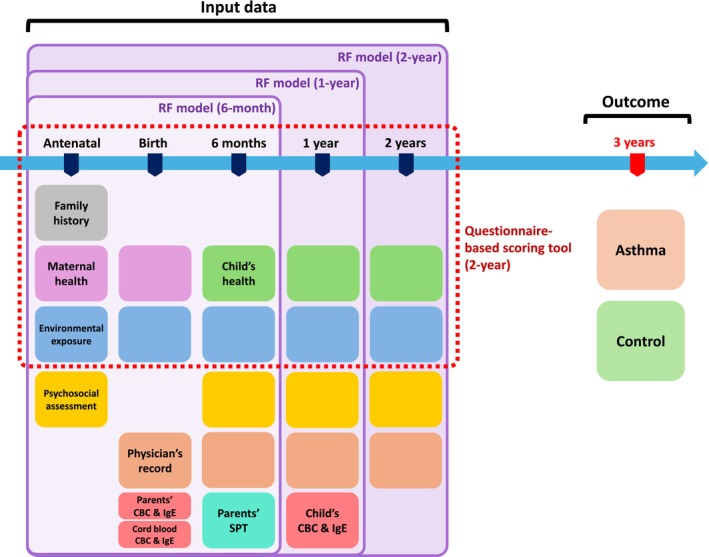
Overview of input data used in COCOA birth cohort and the prediction outcome. This figure illustrates the data components used as model inputs, collected from the antenatal period up to the age of 2 years. Each colored box represents a specific category of data collected at various time points. Family history, maternal health, child's health, and environmental exposure are collected via questionnaires. The datasets used for developing prediction models at different follow‐up periods, as well as the questionnaire‐based model, are indicated. The outcome—the classification into asthma or control groups—was determined at the three‐year follow‐up. CBC, complete blood count; COCOA, COhort for Childhood Origin of Asthma and allergic diseases; IgE, immunoglobulin E; RF, Random Forest; SPT, skin prick test.

To evaluate the predictive value of the data collected during early life and examine how accumulating data up to the age of 2 years influences model performance, three distinct datasets were constructed using variables collected up to 6 months, 1 year, and 2 years (Figure 1). Even when limited to data collected within the first 2 years of life, the COCOA dataset included more than 10,000 variables, including repeated questionnaire responses, physician‐recorded clinical data, and laboratory test results. Given this high dimensionality, we used least absolute shrinkage and selection operator (LASSO) regression for initial variable selection, identifying the most relevant predictors of asthma at the age of 3 years while reducing dataset complexity and minimizing the risk of model overfitting.^18^ The feature selection process was conducted independently for each dataset of different time frames (6 months, 1 year, and 2 years).

### 2.4 Development of the Predictive Model

The Random Forest algorithm was selected to develop a predictive model for the presence of asthma at 3 years of age because this algorithm can effectively manage high‐dimensional data and capture complex interactions among variables.^19^ To optimize the Random Forest model, hyperparameters were tuned using 5‐fold cross‐validation, and the model with the maximized average performance was selected. Following model training, feature importance metrics were calculated for each variable to identify their clinical relevance. In addition, we applied nested 5‐fold cross‐validation separately to the datasets corresponding to the 6‐month, 1‐year, and 2‐year time frames within the development cohort. For each outer fold, hyperparameter tuning was performed on the development set using an inner 5‐fold cross‐validation, and the model performance was assessed on the held‐out fold.

### 2.5 Development of the Questionnaire‐Based Scoring Tool

A separate questionnaire‐based model was developed using data collected up to the age of 2 years. The variables included in this model were limited to family history, demographics, and questionnaire responses on clinical and environmental factors, while explicitly excluding physician‐reported variables, laboratory and allergy test results, and psychosocial survey data. The objective was to develop a scoring tool that can be readily applied in general clinical settings without the need for advanced or specialized evaluations. The features to be included in this scoring system were initially selected using LASSO regression. Further refinement was performed based on predefined exclusion criteria, including a low response rate (<2%), semantic redundancy, and clinical irrelevance. Subsequently, each selected variable was assigned a score based on the rounded values of odds ratios (ORs) derived from logistic regression predicting asthma diagnosis at 3 years of age. The optimal cut‐off point of the scoring tool was identified using the Youden index, which maximizes the sum of sensitivity and specificity.

Additionally, to validate the robustness of our scoring system development method, we directly compared the performance of this tool with that of several ML models. Using the identical set of questionnaire variables used in the scoring tool, we developed predictive models based on Random Forest, Gradient Boosting Machine, and Support Vector Machine algorithms. The performance of this scoring tool in the development set was then compared with the average performance derived from a 5‐fold cross‐validation on each of these ML models. To ensure a fair and robust evaluation, each model was assessed using nested 5‐fold cross‐validation, in which hyperparameter tuning was performed within each training fold using an inner 5‐fold cross‐validation.

### 2.6 Statistical Analysis

All data were presented as mean ± standard deviation for continuous variables and frequency (%) for categorical variables. Differences in continuous variables between the two study groups were compared using an independent two‐sample *t*‐test, whereas categorical variables were compared using the chi‐squared or Fisher's exact test, as appropriate. Model performance was evaluated in the validation dataset using receiver operating characteristic (ROC) curve analysis, with the area under the ROC curve (AUROC) as the primary metric. Additional performance metrics included sensitivity, specificity, positive predictive value (PPV), and negative predictive value (NPV), which were calculated using the Youden index as the cut‐off point. To provide a benchmark for comparison, we also calculated the stringent Asthma Predictive Index (API) using available cohort variables assessed at age 2 years (see Appendix S1).^8^ Data preprocessing and statistical analyses were performed using R software (version 4.4.1; R Foundation for Statistical Computing, Vienna, Austria). Statistical significance was set at *p* < .05.

## 3 RESULTS

### 3.1 Cohort Characteristics

The flow of participants in the study is depicted in Figure S1. Among the 3103 participants initially enrolled in the COCOA study, 181 withdrew consent, leaving a follow‐up cohort of 2922 children. Of these, 2007 children with complete follow‐up data until the age of 3 years were included in this study. The cohort was divided into development (*n* = 1472) and validation (*n* = 535) cohorts. The demographic and baseline characteristics of the development and validation cohorts are presented in Table 1. History of allergic disease diagnoses was classified based on the operational definitions provided in Table S1.

**TABLE 1:** Baseline characteristics of the development and validation cohorts.

Characteristics	Development (*n* = 1472)	Validation (*n* = 535)	*p*
Sex, female, *n* (%)	663 (45.1)	165 (30.8)	<.001
Birth weight, kg (SD)	3.18 (0.42)	3.23 (0.42)	.082
Gestational age at birth, weeks (SD)	39.2 (1.2)	39.1 (2.2)	.301
Caesarean section, *n* (%)	479 (32.5)	139 (26.0)	.006
Family history			
Maternal asthma, *n* (%)	46 (3.1)	21 (3.9)	.458
Maternal other allergic diseases, *n* (%)^a^	765 (52.0)	284 (53.1)	.696
Paternal asthma, *n* (%)	63 (4.3)	31 (5.8)	.193
Paternal other allergic diseases, *n* (%)^a^	686 (46.6)	238 (44.5)	.429
History of symptom or diagnosis at 2 years of age^b^			
Wheezing, *n* (%)	708 (48.1)	266 (49.7)	.554
Allergic rhinitis, *n* (%)	823 (55.9)	336 (62.8)	.007
Atopic dermatitis, *n* (%)	788 (53.5)	312 (58.3)	.064
Food allergy, *n* (%)	210 (14.3)	96 (17.9)	.050
Asthma at 3 years of age, *n* (%)	134 (9.1)	53 (9.9)	.645
No allergic disease by age 3 years of age, *n* (%)	366 (24.9)	123 (23.0)	.420

Abbreviation: SD, standard deviation.

^a^ Other allergic diseases include allergic rhinitis, atopic dermatitis, and food allergy.

^b^ History, determined based on physician's documentation and parental questionnaire responses, was considered positive if either source indicated a positive history.

From the development cohort, 134 participants with asthma at 3 years of age, along with 366 children identified as controls, were included in model development, resulting in a total of 500 participants. The model was validated in an independent validation cohort that included 53 children with asthma and 123 control participants. Baseline characteristics of participants included and excluded from model development and validation are provided in Table S2.

### 3.2 Random Forest Prediction Models

Three prediction models were developed using data collected up to different follow‐up time points. Variables selected using LASSO regression and their corresponding importance metrics for predicting asthma in the 6‐month, 1‐year, and 2‐year models are presented in Tables S3–S5, respectively. Notably, paternal IgE levels and maternal iron supplementation during pregnancy consistently showed high importance across all time points.

**FIGURE 2:**
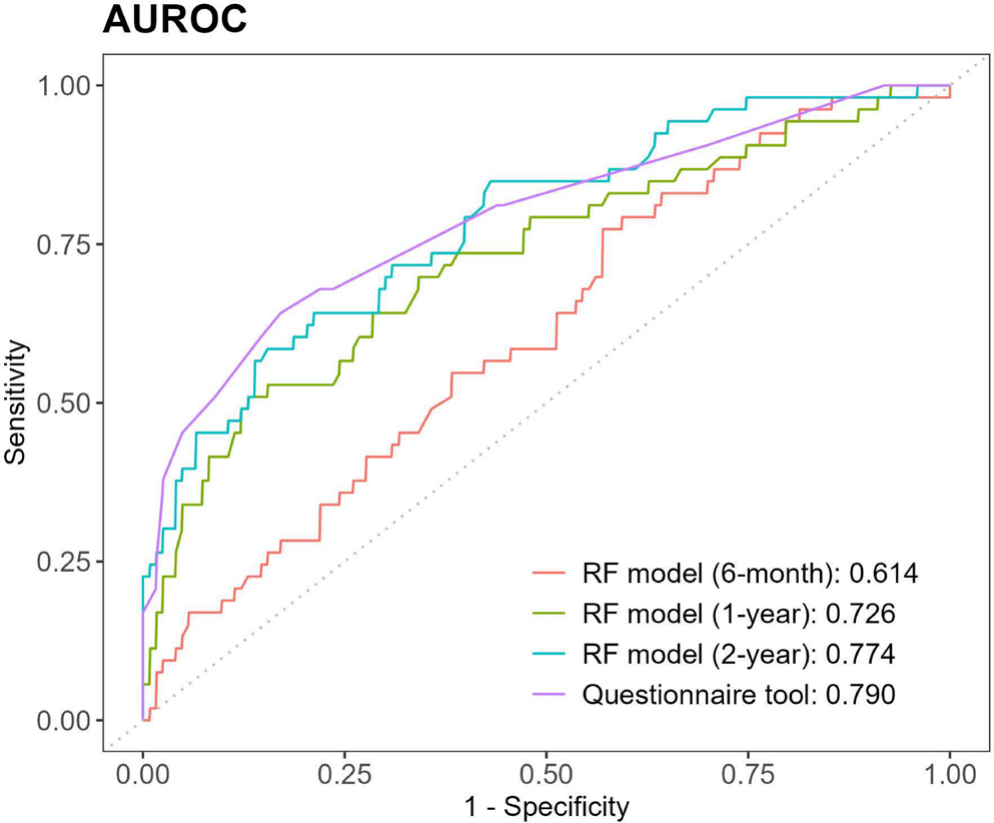
ROC curves of the asthma prediction models. This figure presents ROC curves comparing the performance of ML‐based asthma prediction models developed using data collected up to 6 months, 1 year, and 2 years of age, alongside that of the questionnaire‐based scoring tool. AUC values with corresponding 95% CIs are indicated, demonstrating incremental improvements in model performance with increasing data availability. The questionnaire‐based scoring tool shows a performance similar to that of the 2‐year ML‐based prediction model. AUC, area under the curve; CI, confidence interval; ML, machine learning; ROC, receiver operating characteristic.

The 5‐fold cross‐validation results for each model in the development cohort are summarized in Table S6, and the final model performance in the validation cohort is shown in Table 2. The 6‐month model achieved an AUROC of 0.614, sensitivity of 0.453, specificity of 0.659, PPV of 0.364, and NPV of 0.736. The 1‐year model showed improved performance, with an AUROC of 0.726, sensitivity of 0.604, specificity of 0.699, PPV of 0.464, and NPV of 0.804. As expected, the 2‐year model exhibited the best performance, with an AUROC of 0.774, sensitivity of 0.623, specificity of 0.781, PPV of 0.550, and NPV of 0.828. The ROC curves illustrating the incremental performance improvements across the models are depicted in Figure 2.

**TABLE 2:** Final performance of the asthma prediction models and scoring tool in the validation cohort.

Model	AUROC	Sensitivity	Specificity	PPV	NPV
RF model (6‐month)	0.614	0.453	0.659	0.364	0.736
RF model (1‐year)	0.726	0.604	0.699	0.464	0.804
RF model (2‐year)	0.774	0.623	0.781	0.550	0.828
Questionnaire tool (2‐year)	0.790	0.642	0.829	0.618	0.843
Stringent API	N/A	0.245	0.984	0.867	0.752

Abbreviations: API, Asthma Predictive Index; AUROC, area under the receiver operating characteristics curve; N/A, Not applicable; NPV, negative predictive value; PPV, positive predictive value; RF, Random Forest.

### 3.3 Questionnaire‐Based Scoring Tool

**FIGURE 3:**
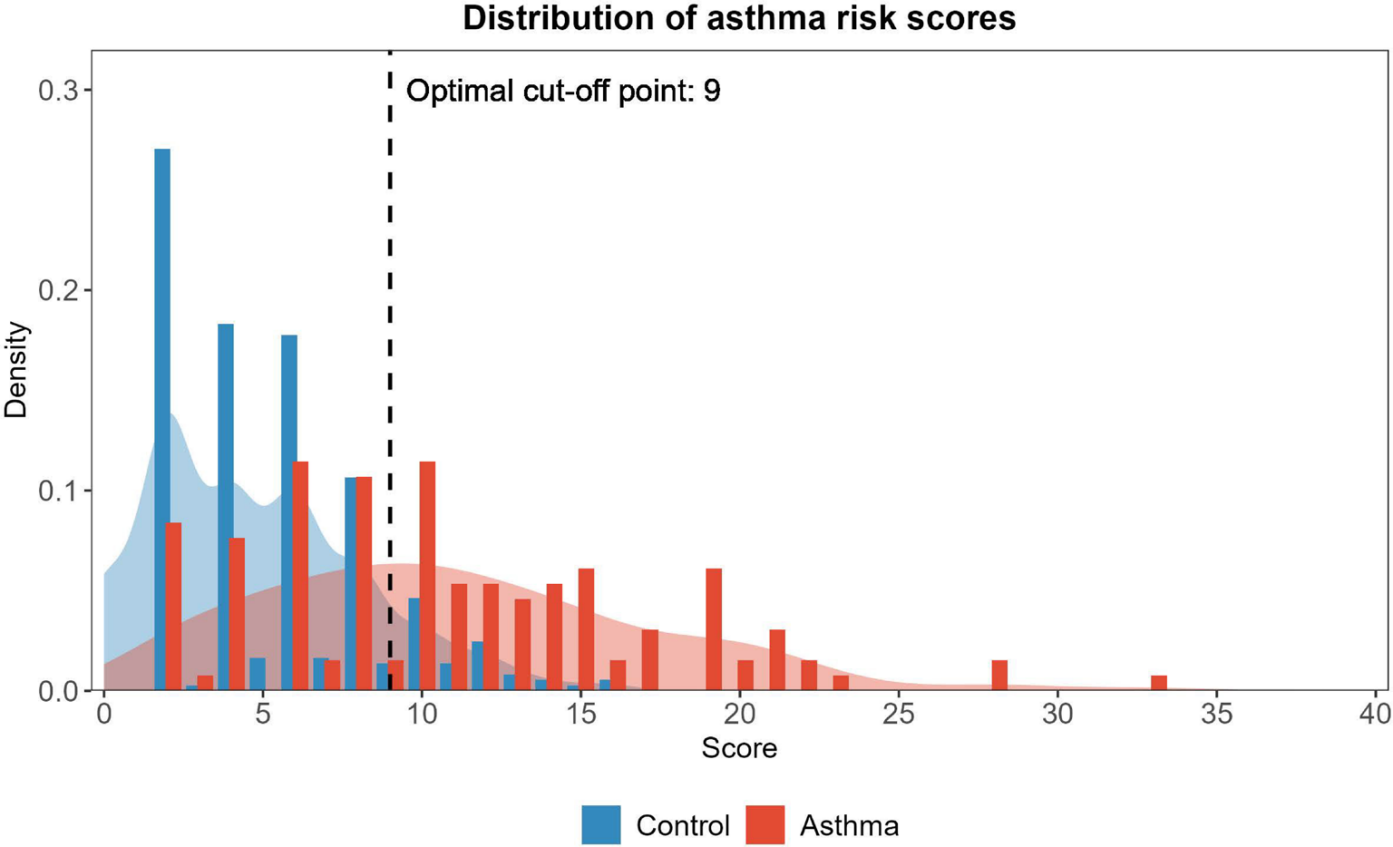
Distribution of questionnaire‐based asthma risk scores and optimal cut‐off point. This figure illustrates the distribution of asthma risk scores derived from the questionnaire‐based scoring tool in the development cohort. Bar plots represent the frequency distribution, and shaded areas indicate density plots for clearer visualization of score distribution patterns. Blue represents the control group, and red represents the group diagnosed with asthma at 3 years of age. The vertical black line denotes the optimal cut‐off point (score = 9), determined using the Youden index to maximize sensitivity and specificity.

Subsequently, a separate model utilizing primarily questionnaire‐based data collected up to the 2‐year follow‐up was developed. An initial set of 22 variables was selected using the LASSO regression. After further selection based on predefined criteria, a final set of 16 variables was used for the model construction. The selected variables and their corresponding ORs derived from the logistic regression analysis are presented in Table 3; not all predictors were individually significant, but each contributed to the overall predictive accuracy of the tool. Notable variables included the diagnosis of food allergy by the age of 2 years (OR 7.218), paternal history of asthma (OR 7.064), and maternal asthma symptoms during pregnancy (OR 4.007). As shown in Table 3, the individual OR values were rounded to create score components for the final scoring system. The distribution of participants in the development cohort according to their score and the optimal cut‐off determined by the Youden index is shown in Figure 3.

**TABLE 3:** Features included in the questionnaire‐based scoring tool and the final scoring system.

Period	Variables	OR	*p*‐Value	Score
Demographics	Male	1.63 (1.00–1.54)	.054	2
	Paternal asthma history	7.06 (1.25–4.38)	.008	7
Prenatal (GA 36 weeks)	Maternal asthma symptoms within 1 year	4.01 (1.13–2.95)	.014	4
	Maternal rhinitis symptoms within 1 year	1.73 (1.05–1.53)	.05	2
	Treatment for epidemic conjunctivitis within 1 year	1.97 (0.74–2.44)	.331	2
	Alcohol during pregnancy	2.33 (1.01–2.05)	.042	2
1 year	Use of moisturizers or medications for atopic dermatitis	3.75 (0.10–30.88)	.694	4
	Eye itching	3.35 (1.16–2.47)	.007	3
	Diagnosis of bronchiolitis within 6 months	2.14 (1.03–1.87)	.030	2
2 years	Symptoms of suspected food allergy	2.91 (1.16–2.19)	.004	3
	Food allergy to nuts	7.22 (1.05–5.31)	.038	7
	Skin rash lasting more than 2 weeks	2.49 (1.17–1.89)	.001	2
	Diagnosis of allergic rhinitis	1.82 (0.92–1.83)	.140	2
	Dry cough during the past 6 months	1.81 (0.92–1.83)	.144	2
	Diagnosis of bronchiolitis or pneumonia within 1 year	3.59 (1.40–2.16)	<.001	4
	Presence of kitchen mold stains	2.19 (0.95–2.08)	.090	2

Abbreviations: GA, gestational age; OR, odds ratio.

To assess whether the scoring tool adequately captured the association between the selected variables and the outcome, its performance was compared to that of multiple ML models generated using the same variables. The performance metrics of both the scoring tool and ML models in the development cohort are presented in Table S7. Given that the scoring tool demonstrated equivalent performance, we proceeded to validate its predictive capability in an independent validation cohort.

The final performance of the scoring tool in the validation cohort is illustrated by the ROC curve shown in Figure 2. Based on the determined cut‐off, the scoring tool achieved an AUROC of 0.799, sensitivity of 0.604, specificity of 0.789, PPV of 0.552, and NPV of 0.822 (Table 2), which was similar to the performance of the 2‐year Random Forest asthma prediction model. Furthermore, the questionnaire‐based tool demonstrated higher sensitivity and a more balanced trade‐off between sensitivity and specificity compared to the original API, which is included in Table 2 for reference.

## 4 DISCUSSION

In this study, we developed a robust ML‐based prediction model for the presence of asthma in preschool age using multidimensional data from the COCOA birth cohort, spanning the antenatal, perinatal, and early childhood periods. In addition, we built a separate questionnaire‐only scoring tool, recognizing the practical constraints in routine clinical settings, where advanced evaluation may not be readily available. This scoring tool had a performance comparable to that of ML models, highlighting its potential utility in real‐world settings. Unlike the API, which prioritizes specificity, our models achieved higher sensitivity while maintaining acceptable accuracy, thus offering a more suitable approach for early risk stratification.

Early prediction of asthma allows targeted stratification and personalized clinical approaches, enabling timely interventions and minimizing unnecessary treatments, ultimately leading to enhanced quality of life and reduced risk of persistent asthma.^3^, ^5^ Numerous studies have previously attempted to predict asthma outcomes at the preschool age, increasingly using ML techniques. For instance, data from the Isle of Wight Birth Cohort collected up to the age of 4 years were utilized to predict asthma at school age, specifically at 10 years of age.^11^ In another study, researchers recruited children aged 3–5 years who had already been diagnosed with asthma and predicted its persistence after the age of 6 years or at a 2‐year follow‐up.^12^ Additionally, data from the Canadian Healthy Infant Longitudinal Development (CHILD) birth cohort, collected up to the age of 4 years, were used to predict early asthma onset by the age of 5 years.^13^ Compared with these earlier studies, our models used a narrower timeframe, specifically data collected only up to the age of 2 years, providing a substantially earlier window for risk prediction. Furthermore, we developed a practical questionnaire‐based scoring tool that facilitates broader clinical applicability without the need for advanced laboratory assessments. Unlike prior models that rely on extended follow‐up data or post‐diagnosis trajectories, our approach enables pre‐emptive clinical decision‐making during a critical early‐life period. Despite the inherent diagnostic uncertainty at 3 years of age, our outcome definition integrated both physician‐confirmed diagnoses and structured parental questionnaires, enhancing sensitivity and ecological validity.

The COCOA dataset provided the largest number of candidate variables with high granularity, even in the questionnaire‐based model, where the variables were more limited. Compared to the CHILD cohort study, which also explored preschool asthma prediction using various time frames from birth cohort data, our ML‐based prediction model and questionnaire‐based scoring tool demonstrated performance capabilities equivalent to those of their models that used data collected up to age two.^13^ The use of LASSO regression with nested cross‐validation in this study ensured robust feature selection and minimized overfitting, contributing to the model stability across validation cohorts. Although ML‐based models offer strong performance, their clinical implementation may be limited by their lack of transparency. Our scoring tool addresses this issue by providing an interpretable framework based on clearly defined questionnaire variables and risk associations. For example, a pediatrician could calculate a score by summing the assigned points from Table 3: a male child (2 points) with allergic rhinitis (2 points) and paternal asthma (7 points) would have a total score of 11, which exceeds the cut‐off of 9 and would classify the child as high risk. In practice, this could prompt closer clinical monitoring and consideration of early interventions, underscoring the tool's practical applicability in routine care.

To the best of our knowledge, this study is the first to leverage birth cohort data to develop a questionnaire‐only prediction scoring tool incorporating prenatal and early‐life environmental factors. This approach holds substantial practical significance as it can be easily applied in general clinical settings to facilitate the early identification of high‐risk children without the need for advanced laboratory testing. Our methodology aligns with previous efforts to create symptom‐based asthma risk stratification tools.^20^, ^21^ Notably, the questionnaire‐only tool demonstrated comparable performance to the ML model, which incorporated parental and child laboratory data. Several factors may explain this finding. The questionnaire tool was based on predictors that captured much of the discriminative signal, whereas the inclusion of additional variables in the ML model may have introduced noise rather than incremental value. In addition, the low case‐to‐variable ratio may have limited the generalizability of complex ML‐based approaches. This finding supports the notion that the addition of advanced allergic biomarkers may provide only minimal incremental value in early asthma prediction.^22^ The methodological framework used in this study may also be applicable to predicting asthma across developmental stages using various age‐specific input variables.

Our predictive models highlighted several clinically relevant risk factors for early asthma. The ML model identified paternal total IgE levels, maternal iron supplementation during pregnancy, and recent bronchiolitis as the most important predictors. In the questionnaire‐based scoring tool, key predictors included parental history of asthma or asthma‐related symptoms, a child's history of tree nut allergy, and recent infections of the lower respiratory tract. Some of these variables, such as family history of asthma and prior respiratory infection, are consistent with predictors commonly reported in previous asthma prediction models.^13^, ^23^ However, several commonly described risk factors, including previous antibiotic use and tobacco smoke exposure, were not selected in our models. It is important to note that feature selection was performed using LASSO regression. Although LASSO regression is a powerful technique for handling high‐dimensional data, it may inadvertently exclude important variables due to their collinearity with other selected features.^18^, ^24^ Therefore, the final selected features should be interpreted as a representative and largely independent set of variables that contribute efficiently to asthma prediction in preschool‐aged children rather than as definitive indicators of causality or underlying mechanisms.

The variables identified as significant predictors in our models aligned well with those of previous studies. First, parental history of asthma, prominently featured in our questionnaire‐based model, is a widely recognized predictor of childhood asthma.^3^, ^6^ Although our ML‐based prediction model specifically highlighted paternal total IgE level, elevated levels of paternal IgE have also been independently associated with an increased risk of childhood asthma in previous studies.^25^, ^26^ Second, recent lower respiratory infections, including bronchiolitis, have consistently been recognized as strong predictors of subsequent asthma development, as evidenced by several observational studies.^27^, ^28^ Third, a child's history of tree nut allergy was identified as another significant predictor in our models, consistent with previous findings that have demonstrated associations of peanut and tree nut allergies with increased asthma risk.^29^, ^30^ Lastly, maternal intake of iron supplements during pregnancy was notably predictive in our study. While previous findings regarding maternal hemoglobin levels and dietary iron intake in relation to childhood allergic outcomes have been somewhat conflicting,^31^, ^32^ trial‐based evidence from Finland has shown that routine iron supplementation during pregnancy was associated with a reduced incidence of physician‐diagnosed asthma in offspring, supporting a potential protective effect.^33^ Our finding is consistent with this evidence, suggesting that maternal iron supplementation may reduce the risk of early‐life asthma. Taken together, these results underscore the potential importance of adequate iron supplementation as part of prenatal care strategies, although further research is needed to confirm causality and to refine clinical recommendations.

This study has several notable strengths. It leveraged data from the COCOA birth cohort, which is characterized by high granularity across multiple early‐life domains. Our methodological framework for developing ML‐based prediction models and an interpretable scoring tool ensures their robustness and generalizability. It also allows scalable application across various developmental time frames. The development of a practical questionnaire‐only scoring tool significantly enhances clinical applicability, facilitating broad implementation for early intervention and personalized asthma management in routine practice. Notably, as one of the few large‐scale prospective birth cohorts based in East Asia, this study contributes unique insights beyond Western‐centric models and may reveal ethnicity‐specific risk patterns relevant to global asthma prevention strategies.

Our study has some limitations. First, the prediction targeted asthma diagnosed at 3 years of age, which is an early developmental stage that may be associated with an uncertain diagnosis. Asthma at this age may include transient rather than persistent wheezing phenotypes, which could affect predictive validity. Therefore, further research with longer follow‐up into school age, including confirmation with lung function testing, will be essential to refine our models and establish their accuracy for later asthma outcomes. Second, many variables in the COCOA study were derived from parental questionnaire responses, introducing potential subjective bias and reducing their reliability. Nonetheless, this approach reflects real‐world clinical scenarios in which detailed history‐taking often depends on parental recall. Third, despite using advanced imputation methods such as MissForest, the substantial percentage of missing data for some variables may have introduced bias or uncertainty in the model predictions. Fourth, the study used temporal validation within COCOA, which may not be representative of the general population. Moreover, our control group was restricted to children with no history of allergic disease. Although this enhanced the clarity of the case–control distinction, it may have reduced the generalizability of our results. In addition, as the models were developed within a Korean cohort, their applicability to other ethnic populations remains uncertain. Future external validation, ideally through international collaborations, will be valuable to confirm the adaptability of our models across diverse populations. Fifth, another limitation is the relatively low case‐to‐variable ratio in our development cohort. We attempted to mitigate this by applying LASSO regression and nested cross‐validation, but future studies with larger case numbers will be important to further strengthen model robustness. Finally, ML‐based models inherently focus on prediction rather than causation, thus limiting insights into the underlying biological and pathophysiological mechanisms. Therefore, further exploration of the causal relationships between the identified predictors and asthma development is required.

In conclusion, we developed an ML‐based prediction model and a practical questionnaire‐based scoring tool for the early prediction of asthma in preschool age, using comprehensive birth cohort data. Both models demonstrated robust performance and identified clinically relevant predictors consistent with the existing literature. Identified predictors, including paternal IgE levels, maternal iron supplementation during pregnancy, parental asthma history, the child's nut allergy, and recent infections of the lower respiratory tract, highlight potential targets for clinical surveillance and preventive strategies. Given the simplicity and scalability of our questionnaire‐based tool, our methodological framework has the potential to develop broadly applicable predictive models suitable for routine clinical practice, facilitating early intervention and personalized management. Nevertheless, further validation in diverse populations and deeper exploration of the causative mechanisms of the identified predictors are essential to enhance the clinical utility of these predictive tools.

## AUTHOR CONTRIBUTIONS

**Chang Hoon Han:** Funding acquisition; writing – original draft; writing – review and editing; software; data curation; validation; visualization; formal analysis; methodology; conceptualization. **Seok‐Jae Heo:** Writing – original draft; writing – review and editing; software; data curation; validation; visualization; formal analysis; methodology; conceptualization. **Haerin Jang:** Resources; data curation; writing – review and editing; investigation. **So‐Yeon Lee:** Resources; data curation; writing – review and editing; investigation. **Ji Soo Park:** Data curation; resources; investigation; writing – review and editing; funding acquisition. **Dong In Suh:** Investigation; funding acquisition; writing – review and editing; data curation; resources. **Youn Ho Shin:** Investigation; funding acquisition; writing – review and editing; data curation; resources. **Jihyun Kim:** Data curation; resources; writing – review and editing; funding acquisition; investigation. **Kangmo Ahn:** Investigation; funding acquisition; writing – review and editing; data curation; resources. **Myung Hyun Sohn:** Data curation; resources; writing – review and editing; investigation; funding acquisition. **Eom Ji Choi:** Investigation; funding acquisition; writing – review and editing; data curation; resources. **Sun Hee Choi:** Data curation; resources; writing – review and editing; investigation; funding acquisition. **Hey‐Sung Baek:** Investigation; funding acquisition; writing – review and editing; data curation; resources. **Soo‐Jong Hong:** Investigation; funding acquisition; writing – review and editing; data curation; resources; project administration; supervision. **Kyung Won Kim:** Data curation; supervision; resources; project administration; writing – review and editing; funding acquisition; investigation; conceptualization; writing – original draft. **Inkyung Jung:** Project administration; writing – review and editing; writing – original draft; supervision; data curation; investigation; methodology. **Soo Yeon Kim:** Writing – original draft; writing – review and editing; conceptualization; investigation; funding acquisition; project administration; data curation; supervision; resources.

## FUNDING INFORMATION

This research was supported by a grant from the MD‐Phd/Medical Scientist Training Program through the Korea Health Industry Development Institute (KHIDI), funded by the Ministry of Health and Welfare, Republic of Korea. This work was also supported by the Research Program funded by the Korea National Institute of Health (2008‐E33030‐00, 2009‐E33033‐00, 2011‐E33021‐00, 2012‐E33012‐00, 2013‐E51003‐00, 2014‐E51004‐00, 2014‐E51004‐01, 2014‐E51004‐02, 2017‐E67002‐00, 2017‐E67002‐01, 2017‐E67002‐02, 2020E670200, 2020E670201, 2020E670202, 2023E120300, 2023E120301, and 2023E120302). The funders had no role in the design and conduct of the study; collection, analysis, and interpretation of data; writing of the report; or decision to submit the paper for publication.

## CONFLICT OF INTEREST STATEMENT

The authors declare no competing financial or non‐financial interests.

## PEER REVIEW

The peer review history for this article is available at https://www.webofscience.com/api/gateway/wos/peer‐review/10.1111/pai.70223.

## Supporting information


Data S1.

## Data Availability

Research data are not shared due to patient confidentiality.
