# Supplementary material for: Machine learning‐based early prediction of asthma in preschoolers: The COCOA birth cohort study
Source: Pediatr Allergy Immunol. 2025 Oct 17;36(10):e70223. doi: 10.1111/pai.70223 (PMC12533341; doi:10.1111/pai.70223)
Supplement: Supplementary file 1 — Data S1. [file PAI-36-e70223-s001.docx]

**SUPPLEMENTARY MATERIALS**

**Table of Contents**

**APPENDIX** [**S1** Application of the stringent Asthma Predictive Index (API) at age 2 2](#_Toc209545721)

[**FIGURE S1** Flow diagram of study participants. 3](#_Toc209545722)

[**TABLE S1** Operational definitions of allergic diseases and control conditions. 4](#_Toc209545723)

[**TABLE S2** Baseline characteristics of included and excluded participants for model development and validation 5](#_Toc209545724)

[**TABLE S3** Variables and their importance in the 6-month prediction model (ordered by importance). 6](#_Toc209545725)

[**TABLE S4** Variables and their importance in the 1-year prediction model (ordered by importance). 7](#_Toc209545726)

[**TABLE S5** Variables and their importance in the 2-year prediction model (ordered by importance). 10](#_Toc209545727)

[**TABLE S6** Cross-validation results for prediction models in the development cohort. 12](#_Toc209545728)

[**TABLE S7** Comparison between the questionnaire-based scoring tool and machine learning models in the development cohort. 13](#_Toc209545729)

[**REFERENCE** 14](#_Toc209545730)

# **SUPPLEMENTARY METHODS** Application of the stringent Asthma Predictive Index (API) at age 2 years

To benchmark the performance of our models against an established clinical tool, we applied the stringent Asthma Predictive Index (API), originally developed in the Tucson Children’s Respiratory Study.^1^ The API estimates the risk of developing persistent asthma in early childhood based on a combination of wheezing frequency and specific clinical risk factors. In our implementation, all variables were assessed using data collected up to age 2 years, to ensure a fair comparison with our machine learning models and questionnaire-based scoring tool, which were also restricted to data from the same time frame. Although the original API was not specifically validated for predicting asthma by age 3, the performance observed in our cohort was broadly comparable to that reported in previous studies, which typically used the API to predict asthma outcomes at later ages, such as school age.^1,2^

We did not apply the modified API (mAPI), as our dataset lacked key components required for its calculation.^2^ Specifically, wheezing frequency was collected via questionnaire and defined as ≥3 episodes per year, rather than the ≥4 threshold used in the mAPI. In addition, sensitization to aeroallergens and food allergens—other than milk and egg—had not yet been assessed in the cohort by age 2.

The stringent API is defined as follows:

| More than 3 episodes of wheezing per year  AND  At least one major criterion or two or more minor criteria | |
| --- | --- |
| Major | Minor |
| Parental history of asthma  Physician-diagnosed atopic dermatitis | Physician-diagnosed allergic rhinitis  Wheezing apart from colds  Blood eosinophilia ≥4% |

# **FIGURE S1** Flow diagram of study participants.

**
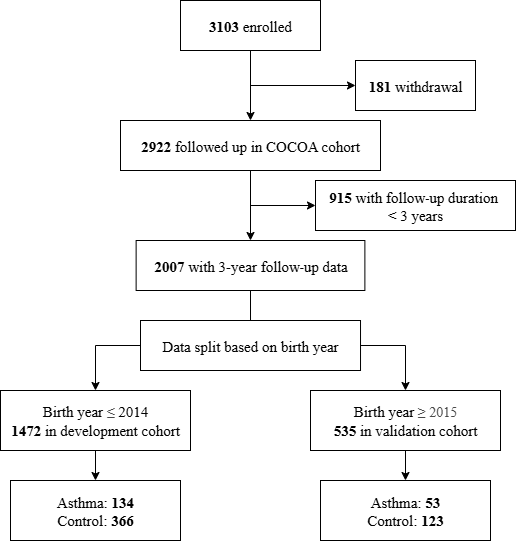
**

COCOA, COhort for Childhood Origin of Asthma and allergic diseases.

# **TABLE S1** Operational definitions of allergic diseases and control conditions.

|  | **Physician’s record** | **Questionnaire response** |
| --- | --- | --- |
| **Asthma**^†^ | **① OR ②** | **① OR ② OR ③** |
|  | **① Diagnosis:**  (Diagnosis of asthma) **OR**  (History of recurrent wheezing) **OR**  (Diagnosis of bronchiolitis **AND** Number of bronchiolitis events >2)  **② Treatment:**  (Treatment of asthma) | **① Symptoms:**  (Wheezing) **AND**  (Number of wheezing events >2)  **② History of diagnosis:**  (History of asthma diagnosis)  **③ History of treatment:**  (History of asthma treatment) |
| **Allergic rhinitis**^†^ | **① OR ②** | **① OR ② OR ③** |
|  | **① Diagnosis:**  (Diagnosis of allergic rhinitis) **OR**  (Diagnosis of allergic conjunctivitis)  **② Treatment:**  (Treatment of allergic rhinitis) | **① Symptom:**  (Sneezing or watery rhinorrhoea) **OR**  (Eye itching)  **② History of diagnosis:**  (History of allergic rhinitis diagnosis)  **③ History of treatment:**  (History of allergic rhinitis treatment) **OR**  (History of allergic conjunctivitis treatment) |
| **Atopic dermatitis**^†^ | **① OR ②** | **① OR ② OR ③** |
|  | **① Diagnosis:**  (Diagnosis of atopic dermatitis)  **② Treatment:**  (Treatment of atopic dermatitis) | **① Symptom:**  (Rash lasting more than 2 weeks)  **② History of diagnosis:**  (History of eczema diagnosis)  **③ History of treatment:**  (History of atopic dermatitis treatment) |
| **Food allergy**^†^ | **①** | **① AND ②** |
|  | **① Diagnosis:**  (Diagnosis of food allergy) | **① History of diagnosis:**  (History of food allergy diagnosis)  **② Dietary restriction:**  (Current restriction of specific foods) |
| **Control**^‡^ | **① AND ②** | **①** |
|  | **① No diagnosis:**  (No diagnosis of any allergic disease) **AND**  (Number of bronchiolitis events **<**3 **OR** NA)  **② No treatment:**  (No treatment of asthma) **AND**  (No treatment of allergic rhinitis) **AND**  (No treatment of atopic dermatitis) | **① No history of diagnosis:**  (No history of asthma diagnosis) **AND**  (No history of allergic rhinitis diagnosis) **AND**  (No history of eczema diagnosis) **AND**  (No history of food allergy diagnosis) |

^†^Cases were defined as participants meeting physician’s report **OR** questionnaire response criteria.

^‡^Controls were defined as participants meeting both physician’s report **AND** questionnaire response criteria.

NA, not assessed.

# **TABLE S2** Baseline characteristics of included and excluded participants for model development and validation

| **Characteristics** | **Development (n=1,472)** | | **P** | **Validation (n=535)** | | **P** |
| --- | --- | --- | --- | --- | --- | --- |
|  | **Included**^†^  **(n=500)** | **Excluded**  **(n=972)** |  | **Included**^†^  **(n=176)** | **Excluded**  **(n=359)** |  |
| Sex, female, n (%) | 218 (43.6) | 445 (45.8) | 0.458 | 61 (34.7) | 104 (29.0) | 0.215 |
| Birth weight, kg (SD) | 3.20 (0.42) | 3.18 (0.42) | 0.336 | 3.27 (0.41) | 3.21 (0.42) | 0.234 |
| Gestational age at birth, weeks (SD) | 39.3 (1.2) | 39.1 (1.2) | 0.337 | 39.2 (1.3) | 39.0 (2.5) | 0.433 |
| Caesarean section, n (%) | 168 (32.5) | 311 (32.0) | 0.573 | 48 (27.3) | 91 (25.3) | 0.710 |
| Family history |  |  |  |  |  |  |
| Maternal asthma, n (%) | 21 (4.2) | 25 (2.6) | 0.123 | 9 (5.1) | 12 (3.3) | 0.451 |
| Maternal other allergic diseases, n (%)^‡^ | 243 (48.6) | 522 (53.7) | 0.072 | 87 (49.4) | 197 (54.9) | 0.274 |
| Paternal asthma, n (%) | 22 (4.4) | 41 (4.2) | 0.978 | 9 (5.1) | 22 (6.1) | 0.783 |
| Paternal other allergic diseases, n (%)^‡^ | 219 (43.8) | 467 (48.0) | 0.135 | 84 (47.7) | 154 (42.9) | 0.335 |
| History of symptom or diagnosis at two years of age^§^ |  |  |  |  |  |  |
| Wheezing, n (%) | 256 (51.2) | 452 (46.5) | 0.098 | 98 (55.7) | 168 (46.8) | 0.066 |
| Allergic rhinitis, n (%) | 236 (47.2) | 587 (60.4) | <0.001 | 101 (57.4) | 235 (65.5) | 0.085 |
| Atopic dermatitis, n (%) | 239 (47.8) | 549 (56.5) | 0.002 | 99 (56.3) | 213 (59.3) | 0.558 |
| Food allergy, n (%) | 45 (9.0) | 165 (17.0) | <0.001 | 30 (17.0) | 66 (18.4) | 0.795 |

^†^Includes both subjects categorized as asthma at three years of age and no allergic disease by age three years of age

^‡^Other allergic diseases include allergic rhinitis, atopic dermatitis, and food allergy.

^§^History, determined based on physician’s documentation and parental questionnaire responses, was considered positive if either source indicated a positive history.

SD, standard deviation.

# **TABLE S3** Variables and their importance in the 6-month prediction model (ordered by importance).

| **Category** | **Description** | **Missing (%)** | **Importance** |
| --- | --- | --- | --- |
| **[Paternal test]** | **Total IgE** | 23.5 | 21.197 |
| **[GA 36 weeks] Maternal questionnaire** | **Duration of iron supplementation regular intake during pregnancy** | 28.4 | 15.580 |
| [6 months] Developmental questionnaire | K-PSI Item 23: ‘Our child doesn’t seem to learn as quickly as other children’. | 23.7 | 8.231 |
| [6 months] Developmental questionnaire | K-PSI Item 45: ‘Our child has more health problems than we expected’. | 23.7 | 8.067 |
| [6 months] Developmental questionnaire | K-EPDS Item 5: ‘I have felt scared or panicky for no very good reason’. | 23.7 | 7.646 |
| [6 months] Developmental questionnaire | K-PSI Item 89: ‘Our child has led to greater expenses than we anticipated’. | 23.7 | 6.908 |
| [GA 36 weeks] Maternal questionnaire | K-STAI Item 2: ‘I feel nervous and restless’. | 20.0 | 6.073 |
| [GA 36 weeks] Maternal questionnaire | K-STAI Item 6: ‘I feel calm’. (reverse-coded) | 20.0 | 6.052 |
| [GA 36 weeks] Maternal questionnaire | Sneezing, runny nose, or nasal congestion symptoms unrelated to influenza in the past 12 months | 3.7 | 4.940 |
| [6 months] Developmental questionnaire | K-ASQ Composite Item 7: ‘Do you have any concerns about your child?’ | 24.3 | 4.408 |
| [Demographics] | Sex | 0 | 4.240 |
| [GA 36 weeks] Maternal questionnaire | Frequently itchy eyes unrelated to epidemic conjunctivitis in the past 12 months | 3.7 | 3.221 |
| [Paternal] Paternal questionnaire | Ever diagnosed with asthma | 3.9 | 2.834 |
| [GA 36 weeks] Maternal questionnaire | Treatment for epidemic conjunctivitis (Apollo eye disease) in the past 12 months | 3.9 | 2.475 |
| [Maternal test] SPT | Tree II allergen | 4.1 | 1.808 |
| [Maternal test] SPT | Tree I allergen | 4.1 | 1.501 |

GA, gestational age; IgE, immunoglobulin E; K-ASQ, Korean-Ages and Stages Questionnaires^3^; K-EPDS, Korean Edinburgh Postnatal Depression Scale^4^; K-PSI, Korean-Parenting Stress Index^5^; K-STAI, Korean State-Trait Anxiety Inventory^6^; SPT, skin prick test.

# **TABLE S4** Variables and their importance in the 1-year prediction model (ordered by importance).

| **Category** | **Description** | **Missing (%)** | **Importance** |
| --- | --- | --- | --- |
| **[Paternal test]** | **Total IgE** | 23.5 | 7.733 |
| **[GA 36 weeks] Maternal questionnaire** | **Duration of iron supplementation regular intake during pregnancy** | 28.4 | 6.363 |
| **[1-year test] CBC** | **Haemoglobin** | 17.1 | 5.989 |
| [1 year] Developmental questionnaire | IBQ-R-Shortform Item 66: ‘When having their hair washed, the baby makes sounds’. | 30.4 | 4.487 |
| [1 year] Developmental questionnaire | IBQ-R-Shortform Item 36: ‘The baby falls asleep within 10 minutes at bedtime’. | 30.4 | 4.484 |
| [6 months] Developmental questionnaire | K-PSI Item 23: ‘Our child doesn’t seem to learn as quickly as other children’. | 23.7 | 4.044 |
| [6 months] Developmental questionnaire | K-EPDS Item 5: ‘I have felt scared or panicky for no very good reason’. | 23.7 | 3.824 |
| [6 months] Developmental questionnaire | K-PSI Item 45: ‘Our child has more health problems than we expected’. | 23.7 | 3.815 |
| [GA 36 weeks] Maternal questionnaire | K-STAI Item 6: ‘I feel calm’. (reverse-coded) | 20.0 | 3.024 |
| [6 months] Developmental questionnaire | K-PSI Item 89: ‘Our child has led to greater expenses than we anticipated’. | 23.7 | 2.980 |
| [1 year] Environmental questionnaire | Diagnosed with bronchiolitis within the past 6 months | 15.1 | 2.851 |
| [GA 36 weeks] Maternal questionnaire | K-STAI Item 2: ‘I feel nervous and restless’. | 20.0 | 2.798 |
| [1 year] Follow-up questionnaire | Allergy treated with medication: Atopic dermatitis | 15.7 | 2.687 |
| [1 year] Developmental questionnaire | K-ASQ Composite Item 6: ‘Has your child been seriously ill or injured in the past few months?’ | 30.6 | 2.539 |
| [GA 36 weeks] Environmental questionnaire | Number of rooms in home | 3.9 | 2.525 |
| [1 year] Follow-up questionnaire | Skin rash lasting more than 2 weeks | 12.9 | 2.343 |
| [GA 36 weeks] Maternal questionnaire | Sneezing, runny nose, or nasal congestion symptoms unrelated to influenza in the past 12 months | 3.7 | 2.28 |
| [1 year] Follow-up questionnaire | Eye itching symptoms | 16.1 | 2.256 |
| [1 year] Developmental questionnaire | IBQ-R-Shortform Item 47: ‘The baby laughs during peek-a-boo games’. | 30.2 | 2.136 |
| [Demographics] | Sex | 0 | 2.099 |
| [GA 36 weeks] Maternal questionnaire | K-STAI Item 3: ‘I feel satisfied with myself’. (reverse-coded) | 20.4 | 2.093 |
| [6 months] Follow-up questionnaire | Dry cough during the past 6 months | 10.0 | 2.053 |
| [1 year] Environmental questionnaire | Mould in the kitchen | 31.0 | 1.908 |
| [1 year] Follow-up questionnaire | Allergy treated with medication: Moisturiser | 15.9 | 1.884 |
| [GA 36 weeks] Maternal questionnaire | Ever diagnosed with epidemic conjunctivitis (Apollo eye disease) | 3.7 | 1.735 |
| [1 year] Environmental questionnaire | Diagnosed with acute enteritis within the past 6 months | 15.5 | 1.696 |
| [Paternal test] SPT | Cat allergen | 17.5 | 1.659 |
| [6 months] Environmental questionnaire | Home remodelling status (current residence) | 10.4 | 1.637 |
| [GA 36 weeks] Maternal questionnaire | Frequently itchy eyes unrelated to epidemic conjunctivitis in the past 12 months | 3.7 | 1.636 |
| [GA 36 weeks] Environmental prenatal questionnaire | Current home wall finish: Wood | 3.9 | 1.488 |
| [1 year] Follow-up questionnaire | Allergy treated with medication: Antihistamine | 15.7 | 1.419 |
| [GA 36 weeks] Maternal questionnaire | Average alcohol intake per occasion | 13.9 | 1.416 |
| [Paternal] Paternal questionnaire | Ever diagnosed with asthma | 3.9 | 1.386 |
| [GA 36 weeks] Maternal questionnaire | Wheezing or whistling sounds in the chest during/after exercise in the past 12 months | 3.9 | 1.38 |
| [GA 36 weeks] Maternal questionnaire | Treatment for epidemic conjunctivitis in the past 12 months | 3.9 | 1.202 |
| [1 year] Developmental questionnaire | K-ASQ Composite Item 3: ‘When you help your child stand, does your child usually place their entire soles flat on the floor?’ | 30.4 | 1.041 |
| [1 year] Follow-up questionnaire | Causative food: Egg | 15.9 | 0.849 |
| [Maternal test] SPT | Tree II allergen | 4.1 | 0.847 |
| [Maternal test] SPT | Tree I allergen | 4.1 | 0.839 |
| [6 months] Dietary questionnaire | Soy-based formula (5 months) | 35.9 | 0.822 |
| [GA 36 weeks] Maternal questionnaire | Timing of wheezing (elementary school period) | 40.8 | 0.725 |

CBC, complete blood count; GA, gestational age; IgE, immunoglobulin E; IBQ-R-Shortform, Infant Behaviour Questionnaire-Revised-Short Form^7^; K-ASQ, Korean-Ages and Stages Questionnaires; K-EPDS, Korean Edinburgh Postnatal Depression Scale; K-PSI, Korean-Parenting Stress Index; K-STAI, Korean State-Trait Anxiety Inventory; SPT, skin prick test.

# **TABLE S5** Variables and their importance in the 2-year prediction model (ordered by importance).

| **Category** | **Description** | **Missing (%)** | **Importance** |
| --- | --- | --- | --- |
| **[Paternal test]** | **Total IgE** | 23.5 | 8.499 |
| **[GA 36 weeks] Maternal questionnaire** | **Duration of iron supplementation regular intake during pregnancy** | 28.4 | 7.081 |
| **[2 years] Environmental questionnaire** | **Diagnosed with bronchiolitis within the past year** | 15.3 | 5.036 |
| [1 year] Developmental questionnaire | IBQ-R-Shortform Item 66: ‘When having their hair washed, the baby makes sounds’. | 30.4 | 4.938 |
| [1 year] Developmental questionnaire | IBQ-R-Shortform Item 36: ‘The baby falls asleep within 10 minutes at bedtime’. | 30.4 | 4.927 |
| [2 years] Developmental questionnaire | K-PSI Item 89: ‘Our child has led to greater expenses than we anticipated’. | 28.0 | 4.853 |
| [6 months] Developmental questionnaire | K-PSI Item 23: ‘Our child doesn’t seem to learn as quickly as other children’. | 23.7 | 4.262 |
| [6 months] Developmental questionnaire | K-PSI Item 45: ‘Our child has more health problems than we expected’. | 23.7 | 3.816 |
| [6 months] Developmental questionnaire | K-EPDS Item 5: ‘I have felt scared or panicky for no very good reason’. | 23.7 | 3.742 |
| [2 years] Follow-up questionnaire | Dry cough during the past 6 months | 10.6 | 3.554 |
| [2 years] Follow-up questionnaire | Skin rash lasting more than 2 weeks | 10.4 | 3.310 |
| [6 months] Developmental questionnaire | Parenting Stress Index Item 89 | 23.7 | 3.191 |
| [GA 36 weeks] Maternal questionnaire | K-STAI Item 6: ‘I feel calm’. (reverse-coded) | 20.0 | 3.112 |
| [GA 36 weeks] Maternal questionnaire | K-STAI Item 2: ‘I feel nervous and restless’. | 20.0 | 2.903 |
| [2 years] Follow-up questionnaire | Food allergy diagnostic test: Blood test | 10.6 | 2.865 |
| [1 year] Environmental questionnaire | Diagnosed with bronchiolitis within the past 6 months | 15.1 | 2.740 |
| [1 year] Developmental questionnaire | K-ASQ Composite Item 6: ‘Recent medical issues?’ | 30.6 | 2.693 |
| [2 years] Follow-up questionnaire | Diagnosed with allergic rhinitis | 12.0 | 2.625 |
| [2 years] Environmental questionnaire | Diagnosed with pneumonia within the past year | 15.5 | 2.408 |
| [GA 36 weeks] Maternal questionnaire | Sneezing, runny nose, or nasal congestion symptoms unrelated to influenza in the past 12 months | 3.7 | 2.335 |
| [1 year] Follow-up questionnaire | Allergy treated with medication: Atopic dermatitis | 15.7 | 2.278 |
| [Demographics] | Sex | 0 | 2.269 |
| [1 year] Follow-up questionnaire | Eye itching symptoms | 16.1 | 2.211 |
| [Paternal test] SPT | Cat allergen | 17.5 | 1.803 |
| [GA 36 weeks] Maternal questionnaire | Frequently itchy eyes unrelated to epidemic conjunctivitis in the past 12 months | 3.7 | 1.757 |
| [2 years] Follow-up questionnaire | Allergy treated with medication: Allergic rhinitis | 10.6 | 1.733 |
| [1 year] Follow-up questionnaire | Allergy treated with medication: Moisturiser | 15.9 | 1.690 |
| [2 years] Environmental questionnaire | Mould in the kitchen (current) | 24.5 | 1.576 |
| [GA 36 weeks] Maternal questionnaire | Received treatment for epidemic conjunctivitis in the past 12 months | 3.9 | 1.554 |
| [GA 36 weeks] Maternal questionnaire | Wheezing or whistling sounds in the chest during/after exercise in the past 12 months | 3.9 | 1.519 |
| [2 years] Follow-up questionnaire | Food allergy: Nuts (walnut, almond, pine nut, etc.) | 10.6 | 1.407 |
| [Paternal] Paternal questionnaire | Ever diagnosed with asthma | 3.9 | 1.305 |
| [GA 36 weeks] Maternal questionnaire | Timing of wheezing (Elementary school period) | 40.8 | 0.922 |
| [Maternal test] SPT | Tree II allergen | 4.1 | 0.870 |
| [Maternal test] SPT | Tree I allergen | 4.1 | 0.794 |
| [2 years] Follow-up questionnaire | Causative food: Beef | 10.6 | 0.564 |

GA, gestational age; IBQ-R-Shortform, Infant Behaviour Questionnaire-Revised-Short Form; K-ASQ, Korean-Ages and Stages Questionnaires; K-EPDS, Korean Edinburgh Postnatal Depression Scale; K-PSI, Korean-Parenting Stress Index; K-STAI, Korean State-Trait Anxiety Inventory; SPT, skin prick test.

# **TABLE S6** Cross-validation results for prediction models in the development cohort.

| **Model** | **Fold** | **AUROC** | **Sensitivity** | **Specificity** | **PPV** | **NPV** |
| --- | --- | --- | --- | --- | --- | --- |
| **6-Month** | 1 | 0.749 | 0.621 | 0.809 | 0.581 | 0.833 |
|  | 2 | 0.773 | 0.423 | 0.859 | 0.524 | 0.803 |
|  | 3 | 0.764 | 0.609 | 0.824 | 0.519 | 0.871 |
|  | 4 | 0.802 | 0.793 | 0.721 | 0.548 | 0.891 |
|  | 5 | 0.725 | 0.536 | 0.739 | 0.455 | 0.797 |
|  | Mean | 0.762 | 0.596 | 0.790 | 0.525 | 0.839 |
| **1-Year** | 1 | 0.791 | 0.621 | 0.779 | 0.546 | 0.828 |
|  | 2 | 0.839 | 0.654 | 0.817 | 0.567 | 0.866 |
|  | 3 | 0.888 | 0.826 | 0.797 | 0.559 | 0.937 |
|  | 4 | 0.915 | 0.862 | 0.779 | 0.625 | 0.930 |
|  | 5 | 0.778 | 0.679 | 0.768 | 0.543 | 0.855 |
|  | Mean | 0.842 | 0.728 | 0.788 | 0.568 | 0.883 |
| **2-Year** | 1 | 0.819 | 0.759 | 0.824 | 0.647 | 0.889 |
|  | 2 | 0.827 | 0.577 | 0.887 | 0.652 | 0.851 |
|  | 3 | 0.870 | 0.739 | 0.824 | 0.567 | 0.910 |
|  | 4 | 0.909 | 0.897 | 0.735 | 0.591 | 0.943 |
|  | 5 | 0.840 | 0.607 | 0.855 | 0.630 | 0.843 |
|  | Mean | 0.853 | 0.716 | 0.825 | 0.617 | 0.887 |

AUROC, area under the receiver operating characteristics curve; PPV, positive predictive value; NPV, negative predictive value.

# **TABLE S7** Comparison between the questionnaire-based scoring tool and machine learning models in the development cohort.

| **Model** | **Fold** | **AUROC** | **Sensitivity** | **Specificity** | **PPV** | **NPV** |
| --- | --- | --- | --- | --- | --- | --- |
| **RF** | 1 | 0.782 | 0.704 | 0.643 | 0.432 | 0.849 |
|  | 2 | 0.832 | 0.710 | 0.803 | 0.629 | 0.855 |
|  | 3 | 0.758 | 0.783 | 0.662 | 0.419 | 0.907 |
|  | 4 | 0.880 | 0.846 | 0.761 | 0.564 | 0.931 |
|  | 5 | 0.753 | 0.714 | 0.493 | 0.364 | 0.810 |
|  | Mean | 0.801 | 0.751 | 0.672 | 0.481 | 0.870 |
| **GBM** | 1 | 0.775 | 0.704 | 0.700 | 0.475 | 0.860 |
|  | 2 | 0.820 | 0.677 | 0.864 | 0.700 | 0.851 |
|  | 3 | 0.768 | 0.739 | 0.730 | 0.460 | 0.900 |
|  | 4 | 0.863 | 0.846 | 0.676 | 0.489 | 0.923 |
|  | 5 | 0.741 | 0.750 | 0.507 | 0.382 | 0.833 |
|  | Mean | 0.793 | 0.743 | 0.695 | 0.501 | 0.873 |
| **SVM** | 1 | 0.767 | 0.704 | 0.700 | 0.475 | 0.860 |
|  | 2 | 0.818 | 0.742 | 0.818 | 0.657 | 0.871 |
|  | 3 | 0.779 | 0.739 | 0.730 | 0.460 | 0.900 |
|  | 4 | 0.869 | 0.808 | 0.803 | 0.600 | 0.919 |
|  | 5 | 0.737 | 0.786 | 0.493 | 0.386 | 0.850 |
|  | Mean | 0.794 | 0.756 | 0.709 | 0.516 | 0.880 |
| **Scoring tool** | | 0.813 | 0.769 | 0.727 | 0.507 | 0.896 |

AUROC, area under the receiver operating characteristics curve; GBM, gradient boosting machine; NPV, negative predictive value; PPV, positive predictive value; RF, Random Forest; SVM, support vector machine.

# **REFERENCE**

1. Castro-Rodriguez JA, Holberg CJ, Wright AL, Martinez FD. A clinical index to define risk of asthma in young children with recurrent wheezing. *Am J Respir Crit Care Med*. Oct 2000;162(4 Pt 1):1403-6. doi:10.1164/ajrccm.162.4.9912111

2. Chang TS, Lemanske RF, Jr., Guilbert TW, et al. Evaluation of the modified asthma predictive index in high-risk preschool children. *J Allergy Clin Immunol Pract*. Mar 2013;1(2):152-6. doi:10.1016/j.jaip.2012.10.008

3. Baik Lin Eun HJC, Sinhae Cho, Jin Kyung Kim, Son Moon Shin, Ji Hoon Lee, Jieun Choi, Young Ah Kim, Kyung Ja Oh. The Appropriateness of the Items of Korean Ages and Stages Questionnaires (K-ASQ) Developmental Screening Test in Korean Infants and Children. *Journal of the Korean Child Neurology Society*. 2014;22(2):39-41.

4. Kim J-I. A Validation Study on the Translated Korean Version of the Edinbergh Postnatal Depression Scale. *Korean Journal of Women Health Nursing*. 2006;12(3):204-209.

5. Kyong-Mee Chung SIL, Changseok Lee. Standardization Study for the Korean Version of Parenting Stress Index(K-PSI). *Korean Journal of Clinical Psychology*. 2008;27(3):689-707.

6. Hahn DW LC, Chon KK. Korean adaptation of Spielberger's STAI (K-STAI). *Korean Journal of Health Psychology*. 1996;1:1-14.

7. Jiyoung Lim Y-JB. Validation Study of the Korean Version of Rothbart’s Infant Behavior Questionnaire-Revised. *Korean Journal of Human Ecology*. 2015;24(4):477-497.
